# Ethics guidelines on COVID-19 triage—an emerging international consensus

**DOI:** 10.1186/s13054-020-02927-1

**Published:** 2020-05-06

**Authors:** Susanne Joebges, Nikola Biller-Andorno

**Affiliations:** Institute of Biomedical Ethics and History of Medicine, University of Zurich, Winterthurerstrasse 30, 8006 Zurich, Switzerland

**Keywords:** COVID-19, Pandemic, Triage, Ethics

## Introduction

COVID-19—classified as a pandemic by the WHO on March 11, 2020—is expected to put tremendous strain on many healthcare systems. Early epidemiological analyses show that compared to the seasonal flu, COVID-19 patients may require ventilation much more frequently [1]. This can lead to a shortage of ventilators and intensive care resources, resulting in limited medical care and death [2]. Whereas some countries have been exposed very early [3], others had the opportunity to prepare for the ethical challenges that emerge when intensive care resources become scarce.

In everyday medical practice, ventilation may be withheld or withdrawn if it is not or no longer indicated or against a patient’s will [4]. In crisis situations, such as pandemics, this practice is superimposed by an additional triaging process. Medical factors of triage recommendations typically contain exclusion criteria, a mortality assessment (e.g., Sequential Organ Failure Assessment (SOFA) score), and a re-evaluation requirement [2]. Beyond the medical aspects, however, triaging unavoidably involves moral choices. The main ethical considerations for making such choices concern equity and maximizing benefits [5, 6]. Other criteria such as considering life stages, rewarding prosocial behavior, or giving priority to the worst off have been subject to long-standing controversy [5, 7, 8].

## Ethics guidelines on COVID-19 triage—a synopsis

Over the past few weeks, a number of triaging guidelines have been issued in various countries, including Italy, Switzerland, Austria, Germany, the UK, and Belgium. The table provides a synopsis of the key aspects that are being covered (Table 1). For the purposes of this synopsis, we have chosen to limit ourselves to guidelines of European countries that are available in English or German (cf. https://prioritiesinhealth.org/guidelines).

**Table 1:** Synposis of key aspects

	Italy	Switzerland	Austria	Germany	UK	Belgium
Issuing body	Italian Society of Anesthesia, Analgesia, Resuscitation and Intensive Care (SIAARTI)	Swiss Academy of Medical Sciences/Swiss Society for Intensive care (SGI)	Austrian Society for Anesthesiology, Reanimation and Intensive Care (OEGARI)	Several intensive care professional associations/Academy for Ethics in Medicine (AEM)	NICE	Belgian Society of Intensive Care Medicine
Equity	All patients (COVID and non-COVID) who require intensive therapy treated according to the same criteria	- All patients requiring intensive therapy treated according to the same criteria - No discrimination - Fair allocation procedures	–	All patients who require intensive therapy treated according to the same criteria	All patients who require intensive therapy—before admission clinical frailty scale (CFS)	All patients evaluated according to the same criteria in order to avoid discrimination
Maximizing benefit	- Probability of survival - Life expectancy - Comorbidities and functional status	- Preserving as many lives as possible - Short-term prognosis is decisive - Protection for health professionals	- Short-term survival - Comorbidity	- Short-term survival - Long-term prognosis	- Frailty - Optimizing critical care bed usage (discuss sharing with other hospitals)	- Medical urgency - Frailty - Comorbidities
Considering age/life span	- Age limit “may ultimately need to be set”	- Age “not in itself” a criterion but affects short-term prognosis - Exclusion > 85 years from admission to ICU (if no ICU beds available, resource management through discontinuation of treatment = stage B)	–	- No (de) prioritization “solely because of biological age”	–	- “Age in itself is not a good criterion to decide on disproportionate care”
Additional criteria	–	- Other criteria such as lottery, first come first served, social utility explicitly rejected	- Goals of care - Indication - First come, first served explicitly rejected	- Indication - Social criteria not permissible	–	- Cognitive impairment
Patient will	+	+	+	+	+	+
Termination of therapy	- Decisions to withhold or withdraw life-sustaining treatments “must always be discussed and shared among the healthcare staff, the patients, and their proxies”	- Staged approach to definition of “ICU treatment no longer indicated” - Change therapy goal	- Futility - Proportionality	- Futility - Therapy goal unrealistic - Patient-centered decision	- Desired critical care treatment goals unrealistic - Document decisions and discussions with patient and family	- Disproportionate care (poor long-term expectations) - Openly discuss decision not to initiate or to withdraw life-sustaining therapies with patients/relatives
Additional recommendations	- Every admission to ICU considered and communicated as an “ICU trial” subject to daily reevaluation - Offer non-ICU bed or palliative care	- Resuscitation “not recommended” (stage B) - Transparent decision-making - Offer palliative care	- Initiate decisions as early as possible - Transparent and (as far as possible) participatory decisions (patients/representatives) - Documentation of reasons for forgone interventions - Palliative sedation in ICU	- Use comorbidities, general frailty, prognostic scores (SOFA) for prioritization - Palliative care	- Discuss risks, benefits, and possible likely outcomes with patients, families, and carers - Use decision support tools (where available) - Discuss DNAR decisions with patient	- Measures to maximize ICU capacity - Advance care planning (e.g., nursing home residents) - No out-of-hospital CPR on “elderly patients” during pandemic
Reevaluation	+	+	+	+	+	+
Who decides?	- Second opinion from Coordination Centers or designated experts in difficult cases	- Interprofessional team when possible - Most senior professional carries responsibility	- Mobile intensive care team - Collegial consultation - Ethics advice, if necessary - Debriefing to avoid PTSD	- Interprofessional team - Where appropriate, clinical ethics - Communication strategy through hospitals - Psychosocial support of teams	- Involving critical care teams in ICU admission decision - Support all healthcare professionals	- 2 to 3 physicians with experiences in respiratory failure in the ICU - Teleconsultation - Psychological support for triaging physicians

All guidelines (Table 1) concur that in a situation of scarcity, COVID and non-COVID patients should be treated equitably according to the same criteria [9–14]. However, no guideline argues in favor of a lottery or a “first come, first served” approach. Rather, prognosis—assessed in accordance with current intensive care standards—is seen as an indispensable precondition for maximizing benefit. There is some difference between the guidelines as to the role of short-term vs. long-term survival. Whereas some guidelines (CH, A) refer to short-term survival only as a key triaging criterion, others either do not specify survival (UK, BE) or explicitly allow for the possibility that long-term prognosis (G) or a reduced lifespan, due to old age or to comorbidities, could affect a patient’s access to a ventilator (I). In Switzerland, an age limit is rejected as a criterion in itself, yet an age of over 85 years is mentioned as an exclusion criterion to admission to the ICU if no free beds are available.

All guidelines cite the will of the patient (as expressed in person, through an advance directive or a legal representative) as guiding treatment choices. Futility is also recognized by all guidelines as a justification to end treatment even against patient will. No preferential treatment for specific subgroups is advocated, except for health staff (CH) with a view to maintaining the workforce. Rather, fair decision-making processes are emphasized as well as good palliative care (I, CH, A, G, BE). Most guidelines (CH, A, G, BE) call in their statements for interprofessional teams to make and document triage decisions fairly and transparently; others (I) require a second opinion in case of uncertainty. All guidelines demand regular re-evaluation of the decisions taken. In recognition of the moral stress that taking these decisions may bring on, all guidelines call for psychosocial support for health professionals.

## Discussion

All guidelines have gone through intense deliberations of national associations and bodies to arrive at very similar recommendations. Respect for the patient’s will, fair distribution, and maximization of benefits based on chance of survival are at the heart of the recently issued triaging guidelines. There is some disagreement as to whether only short-term survival should be considered or if more long-term considerations—life expectancy, possibly in combination with quality of life—should have a place as well. Age limits or the exclusion of other patient groups with reduced long-term survival may be very sensitive from a political and psychological point of view. It might be preferable to strengthen advance care planning, assuming that a significant number of patients with a high likelihood of poor outcomes would not opt for intensive care if other choices, such as good palliative care, were readily available to them.

Guidelines have the potential to reduce the burden on those who need to determine which patient gets access to a scarce resource. To the extent that it is unavoidable that physicians “have to decide who must die and whom (they) shall keep alive” [3], this should not happen without clear criteria that result from a consensus process of professional associations, a team approach to decision-making, and the offer of psychological support [9]. It will be of interest to see if artificial intelligence can play an assistive role in such situations [15].

The allocation of scarce resources has been debated within medical ethics for a long time, and procedural criteria have been defined. In order to claim moral legitimacy, the prioritization process must be transparent, inclusive (allowing for participation of all those who may be affected by decisions resulting from the process), evidence-based, and revisable in the light of new information or arguments [8]. It is encouraging to see that the consultative processes that various national bodies have gone through have yielded similar results. Whereas some differences may be due to contextual factors, the high degree of overlap inspires confidence in the robustness of the core.

Communicating these guidelines well is going to be an important task, particularly when dealing with individual patients and their families. The time constraints in developing the guidelines may have precluded a fully participatory approach, but now that a solid basis exists, it will be important to listen to the voices of all those concerned—health professionals, citizens, and other experts—to see if the status quo can be further amended and improved.

## Data Availability

Not applicable.
